# Physical Activity Levels, Perceived Body Appearance, and Body Functioning in Relation to Perceived Wellbeing Among Adolescents

**DOI:** 10.3389/fspor.2022.830913

**Published:** 2022-03-10

**Authors:** Ann-Christin Sollerhed, Johanna Fransson, JIsabelle Skoog, Pernilla Garmy

**Affiliations:** ^1^Department of Humanities, Faculty of Teacher Education, Kristianstad University, Kristianstad, Sweden; ^2^Department of Nursing and Health Sciences, Faculty of Health Sciences, Kristianstad University, Kristianstad, Sweden; ^3^Department of Health Sciences, Faculty of Medicine, Lund University, Lund, Sweden

**Keywords:** physical activity, body image, body appearance, body functioning, adolescents, wellbeing, health

## Abstract

This study aimed to investigate self-reported physical activity levels, perceived body appearance, and body functioning in relation to perceived wellbeing among adolescents. A cross-sectional survey was performed in four upper secondary schools in one municipality in southern Sweden. Data were obtained from questionnaires completed by 1,491 adolescents (55.4% females; median age 16; range 15–17 years) during school hours. The participation rate was 71.4%. Logistic regression analyses were carried out with wellbeing as the dependent variable. The independent variables included gender, perceived family financial situation, perceived body appearance, perceived body function, and physical activity level. Perceived positive wellbeing was associated with being satisfied with their body's appearance (OR 3.4; CI 2.6–4.4) and function (OR 3.1; CI 2.2–4.2), being physically active three or more times per week (OR 1.5; CI 1.1–2.0), and a good perceived family financial situation (OR 3.3; CI 1.6–6.7). Gender was not significantly associated with wellbeing. A positive body image, which include both body appearance and body function, and high physical activity levels were significantly associated with wellbeing in adolescents, corroborating the importance of promoting physical activity among younger populations.

## Introduction

According to the World Health Organization (WHO), mental health “is a state of wellbeing in which the individual realizes his or her own abilities, can cope with the normal stresses of life, can work productively and fruitfully, and is able to make a contribution to his or her community” (World Health Organization, 2018). Adolescent mental health problems are a global public health concern accompanied by a growing disease burden (Whiteford et al., 2013). Mental disorders are estimated to affect 10–20% of children and adolescents worldwide, resulting in short- and long-term consequences that include school disengagement and poor quality of life (Kieling et al., 2011). One in five adolescents are reported to have a mental illness that will persist into adulthood (Kessler et al., 2005) that imposes high costs for society (Suhrcke et al., 2008). Emerging evidence suggests that primary prevention can address some mental health problems and improve the overall mental wellbeing of children and adolescents (Kieling et al., 2011). Given the pervasiveness of mental health disorders in adolescents, importance must be placed on promoting good mental health in this population.

Adolescence is an important period for the development of good health and wellbeing in adulthood (Bluth et al., 2017). Physical activity (PA) is often suggested as a method to improve wellbeing in young people (Biddle and Asare, 2011; Ekkekakis, 2013). Although the physical and psychological benefits of PA are well-established, PA decreases during adolescence (Kemper et al., 2001) and large numbers of adolescents are physically inactive or sedentary (de Moraes et al., 2013). In addition low levels of PA are shown to be independently associated with diminished psychological wellbeing among adolescents (Ussher et al., 2007). Furthermore, cognitive functioning, depression, and self-esteem were found to be associated with PA (Biddle et al., 2019). Self-esteem is considered a key indicator of mental health and as a construct includes emotional stability and subjective wellbeing (Lindwall et al., 2014). Adolescents are recommended to engage in moderate to vigorous physical activity (MVPA) for at least 60 min every day (World Health Organization, 2020); however, 81% of the world's adolescents fail to reach this target (World Health Organization, 2018). Similar results are seen in Sweden, where 87% of adolescent males and 91% of adolescent females do not reach the WHO's recommendations (Public Health Agency of Sweden, 2019). Approximately 10–15% of adolescents were shown to be engaging in MVPA on a regular basis, with females being less active than males (Ekelund et al., 2012; Khan et al., 2015; Chzhen et al., 2018). Over the past decades, PA levels have decreased, and sedentary behavior has increased among adolescents (Tremblay et al., 2011; Nyström et al., 2018). Further, at the age of 15, ~75% of waking time is spent as inactive and sedentary (Public Health Agency of Sweden, 2019).

The 2017–2018 Health Behavior in School-aged Children (HBSC) study found that 66% of adolescent females and 85% of adolescent males aged 15 years in Sweden rated their wellbeing as high or very high (Public Health Agency of Sweden, 2019). This result indicates that there are more adolescent males than adolescent females who report positive wellbeing in Sweden. This discrepancy is also seen in HBSC contributions from other countries (Inchley et al., 2020). In Norway, France, and Italy, adolescent females rated their wellbeing lower than that of adolescent males (Bonsergent et al., 2012; Petracci and Cavrini, 2013; Bjørnsen et al., 2019). Additionally, wellbeing tends to decrease with increasing age (Inchley et al., 2020). In France, a study was conducted with 5,226 adolescents aged 14–18 years in which the results showed less wellbeing in adolescent females and adolescent males in late adolescence as compared to early adolescence (Bonsergent et al., 2012).

Good wellbeing was significantly associated with regular PA (Petracci and Cavrini, 2013; McMahon et al., 2017) and with several health benefits (Janssen and LeBlanc, 2010; Warburton and Bredin, 2017; Rodriguez-Ayllon et al., 2019) and a positive body image (Griffiths et al., 2017; Ra and Cho, 2017). Body image includes the feelings that an individual experiences about his or her body (Slade, 1988; Hosseini and Padhy, 2021). Body image is defined as the internal, subjective representations of physical appearance and bodily experience that encompasses perception of both body appearance and body functioning (Cash and Pruzinsky, 1990). It also includes an attitudinal component that reflects the degree to which individuals are satisfied with body appearance and functioning, and involves how one sees themselves (Grogan, 2021). Body image can be deconstructed into three components: the body's objective features (weight, size, body shape), how the individual experiences his or her body based on its appearance (satisfied, dissatisfied), and how the body functions in daily life (movement, fitness) (Hosseini and Padhy, 2021). However, previous studies do often not take into consideration the different dimensions of body image (Fenton et al., 2010). There is therefore a need for studies that distinguish between body appearance and body function.

For many individuals, appearance reaches a new degree of importance during adolescence (Foley Davelaar, 2021). As the body develops with age, increased concerns about weight and body appearance may emerge (Ren et al., 2018). Generally, female and male adolescents tend to hold a more negative view of their bodies during adolescence than during childhood. According to the Swedish contribution of the HBSC study, the majority of adolescents in Sweden were quite satisfied with the appearance of their bodies (Public Health Agency of Sweden, 2019). More adolescent males than adolescent females thought they were at a normal weight weighed, while more females thought they were overweight (Public Health Agency of Sweden, 2019). Overall, more adolescent females reported holding a negative body image compared to adolescent males (Lawler and Nixon, 2011; Griffiths et al., 2017; Ren et al., 2018), and more females than males (80 vs. 55%, respectively) aged 16 years wanted to change a feature on their body (Lawler and Nixon, 2011). A negative body image was affected by a poor socioeconomic status (SES) (Mikkilä et al., 2003), pressure from friends, and being criticized for one's appearance (Lawler and Nixon, 2011). Furthermore, depression, low self-esteem, and a high body mass index (BMI) were significantly associated with a negative body image among female adolescents (Ganesan et al., 2018). Studies have found that physical self-concepts predict behaviors important for wellbeing, such as PA, dietary behavior and self-esteem (Crocker et al., 2006). Physical self-concepts have been shown to be an even better predictor of behavior than characteristics such as height and weight (Fox, 1997). A recent study among adolescents aged 13–15 in Sweden (Sollerhed et al., 2021) found that good subjective health was associated with good wellbeing in school, good family financial situation, positive body image, and high physical activity levels. Further studies on self-concepts such as body image and PA behavior in relation to wellbeing among adolescents in Sweden are warranted.

Given that previous studies show that many adolescents report poor wellbeing, low PA levels, and negative self-concepts such as poor body image and body anxiety, these observations lead to questions as to how these factors are associated in a population of adolescents in Sweden. Therefore, the purpose of this study is to investigate self-reported PA levels, perceived body appearance, and body functioning in relation to perceived wellbeing among male and female 16-year-old adolescents.

## Materials and Methods

This study used quantitative data from a larger research project (ISRCTN17006300). The study was approved by the Regional Ethical Review Board (EPN 2015/113) and conducted in accordance with the Declaration of Helsinki. Participation in the study was voluntary, and all participants and their parents or legal guardians received both oral and written information about the study. This cross-sectional study was performed in four upper secondary schools in a municipality in southern Sweden with approximately 100,000 inhabitants. Five schools were invited to participate, and one declined. The four participating schools were situated in urban areas, but the students who attended these schools came from both urban and rural areas. One was a private school and three were public schools. The study utilized online questionnaires that were completed during school hours in a classroom with a teacher present. Data collection took place between November 2017 and June 2019. The questionnaire used in our study was found to be reliable for our age group (Sollerhed, 2006).

All students in their first year at the four participating upper secondary schools were asked to participate in the study. The median age of the students was 16 years (range: 15–17 years). Of the 2,089 total respondents, 1,491 (43.7% males, 55.4% females) participated in the study and completed the entire questionnaire, representing a response rate of 71.4%. Participation was voluntary and could be withdrawn without question at any time; however, the age and gender of students who withdrew from the study did not significantly differ from those of participants.

### Statistical Analysis

The online questionnaire included questions about PA, body image (perceived body appearance and body functioning), perceived wellbeing, and perceived family finances. SPSS Statistics v.25 software was used to produce both descriptive and analytical statistics. Data were first analyzed using descriptive statistics with frequencies and percentages. Chi-square tests were used to investigate the associations between perceived wellbeing and the independent variables of body appearance, body functioning, PA, gender, and family financial situation. Subjective health has been shown to be affected by gender and SES (Operario et al., 2004; Marmot and Wilkinson, 2005; Michel et al., 2009) and therefore, these variables were included in our analysis. The relationships between perceived wellbeing and the independent variables of sex, body appearance, body functioning, PA, and perceived family finances were investigated by bivariate analysis. Finally, multiple logistic regression was used to examine whether perceived body appearance, body functioning, PA, gender and perceived family finances had any significant effect on the likelihood of observing positive perceived wellbeing among adolescents. All independent variables were included in a single model, without any additional variable selection (method “enter” in SPSS). The rationale for using a logistic regression analysis is that it allows us to see the effects of variables after adjusting for other variables (e.g., perceived body appearance). This helps us verify that the associations seen in the bivariate analysis aren't due to the influence of other variables.

The response options were collapsed to facilitate data interpretation. For example, the question “How are you most of the time?, ” with its answer options “Very good” and “Quite good” have been recoded into one alternative answer (good wellbeing); likewise with the answer options “Neither good nor bad”, “Pretty bad, ” and “Very bad” (poor wellbeing). The answer option “Do not know” to the question of family financial situation had no natural distribution to the other answer alternatives and was, therefore, not dichotomized. Coding of response options is shown in Table 1. The significance level was set at *p* < 0.05.

**Table 1:** Variables included in the logistic regression with wellbeing as the dependent variable.

**Item**	**Response options in questions**	**Coding**
**Wellbeing**	5 categories	Good wellbeing (1–2)
“How are you most of the time?”	Very good (1) → Very bad (5)	Poor wellbeing (3–5)
**Body appearence**	4 categories	Satisfied (1–2)
“How satisfied are you with your body appearance?”	Yes, completely satisfied (1) → No, not satisfied at all (4)	Not satisfied (3–4)
**Body function**	4 categories	Satisfied (1–2)
“How satisfied are you with how your body works?”	Yes, completely satisfied (1) → No, not satisfied at all (4)	Not satisfied (3–4)
**Physical activity**	7 categories	PA ≥3 times/week (6–7)
“How often do you exercise in your free time for at Least half an hour so that you become short of breath And sweaty?!”	Never (1) → Regularly 4 times or more/week (7)	PA 2 times/week (5)
		PA never, seldom, or once a week (1–4)
**Perceived family financial situation**	5 categories	Good (1–2)
“How well-off do you think your family is?”	Very good (1) → Very bad (5)	Average (3)
		Not so good (4–5)

## Results

As shown in Table 2, the majority of the adolescents rated their wellbeing as good (68%), while 74% perceived their family financial situation as good. The majority of respondents were satisfied with their body appearance (68%) and their body functions (83%), and 52% of respondents reported engaging in PA for at least 30 min three or more times per week. A larger percentage of adolescent females in our sample rated their wellbeing as good relative to adolescent males (71 and 64%, respectively; *p* = 0.003). Furthermore, more adolescent females were satisfied with their body appearance (72%) and body function (85%) compared to adolescent males (63 and 80%, respectively; Table 2).

**Table 2:** Description of wellbeing, body appearance, body function, physical activity, and family financial situation in male and female adolescents (*n* = 1,491).

	**Total, *n* (%)**	**Males, *n* (%)**	**Females, *n* (%)**	***P^a^***
**Wellbeing**	1,458 (97.8)	642 (98.5)	816 (98.8)	0.003
Very good	278 (19.0)	112 (17.4)	166 (20.3)	
Quite good	713 (48.9)	300 (46.7)	413 (50.6)	
Neither good nor bad	327 (22.5)	145 (22.6)	182 (22.3)	
Quite bad	114 (7.8)	70 (10.9)	44 (5.4)	
Very bad	26 (1.8)	15 (2.4)	11 (1.4)	
Missing data	33 (2.2)	10 (1.5)	10 (1.2)	
**Body appearence**	1,461 (98.0)	641 (98.3)	820 (99.3)	<0.0001
Yes, completly satisfied	184 (12.6)	78 (12.2)	106 (12.9)	
Yes, quite satisfied	812 (55.6)	328 (51.2)	484 (59.0)	
No, not that satisfied	340 (23.3)	155 (24.1)	185 (22.6)	
No, not satisfied at all	125 (8.5)	80 (12.5)	45 (5.5)	
Missing data	30 (2.0)	11 (1.7)	6 (0.7)	
**Body function**	1,453 (97.4)	639 (98.0)	814 (98.5)	0.015
Yes, completly satisfied	417 (28.7)	158 (24.7)	259 (31.8)	
Yes, quite satisfied	784 (54.0)	351 (54.9)	433 (53.2)	
No, not that satisfied	200 (13.7)	99 (15.5)	101 (12.4)	
No, not satisfied at all	52 (3.6)	31 (4.9)	21 (2.6)	
Missing data	38 (2.6)	13 (2.0)	12 (1.5)	
**Physical activity**	1,470 (98.6)	645 (98.9)	825 (99.9)	0.008
Regularly ≥4 times/week	456 (31.0)	190 (29.5)	266 (32.2)	
Regularly 3 times/week	307 (20.9)	132 (20.5)	175 (21.2)	
Regularly 2 times/week	243 (16.5)	87 (13.5)	156 (18.9)	
Regularly 1 time/week	198 (13.5)	98 (15.2)	100 (12.1)	
Sometime every month	171 (11.6)	88 (13.6)	83 (10.1)	
Sometime every year	55 (3.8)	28 (4.3)	27 (3.3)	
Never	40 (2.7)	22 (3.4)	18 (2.2)	
Missing data	21 (1.4)	7 (1.1)	1 (0.1)	
**Perceived family financial situation**	1,470 (98.6)	647 (99.2)	823 (99.6)	0.458
Very good	557 (37.9)	230 (35.5)	327 (39.7)	
Quite good	523 (35.6)	234 (36.2)	289 (35.1)	
Average	326 (22.1)	147 (22.7)	179 (21.7)	
Not so good	34 (2.3)	22 (3.4)	12 (1.5)	
Not good at all	7 (0.5)	3 (0.5)	4 (0.5)	
Do not know	23 (1.6)	11 (1.7)	12 (1.5)	
Missing data	21 (1.4)	5 (0.8)	3 (0.4)	

a *Chi-squared test*.

The results of the bivariate analysis showed a significant association between perceived positive wellbeing and being satisfied with one's body appearance and body function among both adolescent males and females (*p* < 0.0001). Perceived positive wellbeing was also associated with being physically active three or more times per week in both male and female adolescents (*p* < 0.0001). An association between perceived positive wellbeing and a perceived good financial situation was also found among adolescent males (*p* < 0.0001) and females (*p* = 0.011; Table 3).

**Table 3:** Relationship between wellbeing, body appearance, body function, physical activity, and family financial situation in male and female adolescents (*n* = 1,491).

	**Males, *n* (%)**			**Females, *n* (%)**		
	**Good wellbeing**	**Poor wellbeing**	p^*^	**Good wellbeing**	**Poor wellbeing**	p^*^
**Body appearence**			<0.0001			<0.0001
Satisfied	311 (76.2)	91 (40.1)		469 (81.4)	116 (49.4)	
Not satisfied	97 (23.8)	136 (59.9)		107 (18.6)	119 (50.6)	
**Body function**			<0.0001			<0.0001
Satisfied	364 (88.8)	141 (62.9)		527 (92.1)	159 (67.4)	
Not satisfied	46 (11.2)	83 (37.1)		45 (7.9)	77 (32.6)	
**Physical activity**			<0.0001			<0.0001
Regularly ≥3 times/week	231 (56.3)	88 (38.4)		333 (57.6)	104 (43.9)	
Regularly 2 times/week	55 (13.4)	32 (14.0)		111 (19.2)	43 (18.1)	
Never, seldom, or once a week	124 (30.2)	109 (47.6)		134 (23.2)	90 (38.0)	
**Perceived family financial situation**			<0.0001			0.011
Good	329 (81.4)	132 (58.9)		449 (78.5)	159 (69.4)	
Average	68 (16.8)	75 (33.5)		115 (20.1)	62 (27.1)	
Not so good	7 (1.7)	17 (7.6)		8 (1.4)	8 (3.4)	

* *Chi-squared test*.

*Missing data: <3.0%*.

The results from the multiple logistic regression analysis showed that perceived positive wellbeing was associated with being satisfied with one's body appearance (OR 3.4; CI 2.6–4.4) and body function (OR 3.1; CI 2.2–4.2), engaging in PA three or more times per week (OR 1.5; CI 1.1–2.0), and perceived good family financial situation (OR 3.3; CI 1.6–6.7). Gender was not significantly associated with wellbeing (Table 4).

**Table 4:** Variables associated with good wellbeing in a multiple logistic regression analysis (*n* = 1,491).

**Variable**	***B***	***SE***	**χ^2^**	***p***	***OR***	**95% CI**
(Intercept)	−2.69	0.60	20.27	<0.001	–	–
Sex (male) reference	–	–	–	–	–	–
Sex (female)	0.57	0.48	1.40	0.236	1.76	[0.69, 4.49]
Economy (poor) reference	–	–	–	–	–	–
Economy (average economic situation)	0.69	0.38	3.32	0.069	2.00	[0.95, 4.21]
Economy (good economic situation)	1.19	0.37	10.45	0.001	3.27	[1.59, 6.72]
PA (once a week or less) reference	–	–	–	–	–	–
PA (twice a week)	0.23	0.19	1.53	0.217	1.26	[0.87, 1.83]
PA (three times a week or more)	0.41	0.14	8.32	0.004	1.51	[1.14, 1.99]
Body appearance (not satisfied) reference	–	–	–	–	–	–
Body appearance (satisfied)	1.22	0.13	85.11	<0.001	3.40	[2.62, 4.41]
Body function (not satisfied) reference	–	–	–	–	–	–
Body function (satisfied)	1.12	0.16	48.65	<0.001	3.07	[2.24, 4.21]

*χ^2^(8) = 262.44, p < 0.001, Hosmer–Lemeshow, p = 0.510, Nagelkerke R^2^ = 0.236. Variance Inflation Factors (VIFs) were calculated to detect the presence of multicollinearity between independent variables. All independent variables in the regression model have VIFs of 1.01–1.06*.

## Discussion

The main findings of our study revealed that PA level, perceived body appearance, and perceived body function were each significantly associated with perceived wellbeing. A greater number of adolescents who were physically active three or more times per week perceived their wellbeing as positive relative to less active or inactive adolescents. Other studies have reported that lower PA levels are significantly associated with worse mental wellbeing (Kirkcaldy et al., 2002; Brodersen et al., 2005), which is in accordance with the results of our study. Given the salutogenic approach used in our study, we focused on positive wellbeing instead of poor wellbeing; however, the pathogenic side of the phenomenon is also shown: low PA levels were associated with poor wellbeing. Similar results were shown in previous studies (Ussher et al., 2007; Petracci and Cavrini, 2013; Ho et al., 2015; McMahon et al., 2017). High PA levels reduced symptoms of depression and anxiety in adolescents (Bell et al., 2019). Thus, primary prevention such as PA can improve overall mental wellbeing of children and youth in different ways, which is generally considered a more appealing treatment than psychotropic medications.

Mental health difficulties affect 10–20% of children and adolescents worldwide (Kieling et al., 2011). At the same time a large proportion of adolescents have low PA levels or are sedentary (World Health Organization, 2018). Moreover, evidence shows that a substantial proportion of mental health problems in adults originate early in life (Kessler et al., 2007). Thus, efforts are urgently needed to reduce the burden of mental health difficulties in adults by detecting and treating these difficulties as early in life as possible. PA is an attractive intervention to this end, because it is a low-cost, non-pharmacological option with few deleterious effects or costs on society. A more resilient mindset appears to be developed among physically active children and adolescents, which may strengthen problem-solving skills that can further enhance their resilience (Edward, 2005).

A greater percentage of the physically active adolescents in our study rated their body functions as good relative to less active adolescents, which may indicate that physically fit adolescents rely more on their movement skills and fitness. They may interpret their high stamina and strength as sufficient to perform regular PA and to help them cope with daily stressors, which increase resilience, self-efficacy, and wellbeing (Edward, 2005). Improving cardiorespiratory fitness has been shown to be an important interventional strategy to promote psychological wellbeing in children (Chen et al., 2021).

In our study, the adolescents who were satisfied with their body functions appear to be in a positive development spiral. They perceive their body function positively, with ability to engage in PA, which in turn is related to wellbeing. Higher levels of regular PA were shown to be associated with better self-perceived health status and quality of life in both adults (Anokye et al., 2012), and youth (Marker et al., 2018). Furthermore, a high percentage of adolescents in our study who reported a positive body image (both body appearance and function) estimated their wellbeing as good, which is in accordance with results from previous studies (Griffiths et al., 2017; Ra and Cho, 2017). Body image, which is defined as the internal subjective perceptions of both body appearance and body functioning (Cash and Pruzinsky, 1990), also includes attitudinal components that involve how one perceives themselves (Grogan, 2021) and experiences appearance and body functions in daily life (Hosseini and Padhy, 2021), which are all vital to wellbeing. The relationship between body image and wellbeing (Gillen, 2015), between body image and PA (Kantanista et al., 2015), and between PA and wellbeing (Biddle and Asare, 2011; Ekkekakis, 2013; Chen et al., 2021) indicate that school and health care providers should encourage PA and positive body image among young persons to improve wellbeing and yield potential health benefits.

The results of the study showed that a perceived good family financial situation was significantly associated with good wellbeing, which has been shown in previous studies. Family financial status can influence the degree to which young people engage in PA (Kirby et al., 2013). SES and financial situation have been shown to provide more opportunities for organized PA in leisure time (Kirby et al., 2013) and is associated with wellbeing (Stalsberg and Pedersen, 2010; Plenty and Mood, 2016). Other studies showed that adolescent's PA was associated with their father's SES and education. However, these associations with SES were weaker than with the fathers' own PA level (Yang et al., 1996). Participation in PA by children and adolescents is greater in families with active parents than in families with less physically active parents (Moore et al., 1991; Yang et al., 1996; Trost et al., 2003; Ornelas et al., 2007). Parents play a large role in determining what type of PA their children engage in and which financial resources they have available, while parental support, modeling, and shared activities appear equally as important or even more important as financial status in determining the type of PA in which their children engage (Gustafson and Rhodes, 2006).

Results from our logistic regression analysis showed that gender was not significantly associated with wellbeing. However, the results showed that more adolescent females than males rated their wellbeing as very good or quite good (71 vs. 64%, *p* = 0.003). This result differs from previous studies, where more adolescent males than females rate their wellbeing as good (Bonsergent et al., 2012; Petracci and Cavrini, 2013; Bjørnsen et al., 2019). Furthermore, more adolescent females than males in this study reported regularly engaging in PA three or more times per week and reported a positive body image, which contradicts the results of other studies (Public Health Agency of Sweden, 2019; Steene-Johannessen et al., 2020). The explanation for our finding that adolescent females were more physically active than adolescent males is unclear. The results may indicate a skewness in the sample of female adolescents, who were as physically active as their male counterparts—and at times slightly more physically active—and their PA level was associated with a high self-reported wellbeing. Furthermore, this sample of physically active females can be seen as a good example of non-specific gender behavior that can serve as a model for improved wellbeing, since PA is suggested to improve wellbeing in young people (Biddle and Asare, 2011; Ekkekakis, 2013) and to improve body image (Vocks et al., 2009).

The present study showed that a positive body image was significantly associated with good wellbeing, which corroborates the results of previous studies (Griffiths et al., 2017; Ra and Cho, 2017). However, approximately one-third of adolescents in our study were dissatisfied with their body appearance. Social media can be a contributing factor to body image, as adolescents are regularly exposed to various social media (Spurr et al., 2013) that portrays a slim body as ideal. Both adolescent males and adolescent females are affected by social media (Keles et al., 2020). Adolescent males largely strive for a more muscular body, while adolescent females predominantly desire a lean body (Spurr et al., 2013). Other factors that can negatively affect body image are stress, depression (El Ansari et al., 2014), and poorer financial conditions (Ren et al., 2018). The present study showed that adolescent females were more satisfied with their body's appearance and its function than were adolescent males, which differs from previous research. For example, a Dublin study found that 40% of adolescent males and 19% of adolescent females were satisfied with the appearance of their bodies (Lawler and Nixon, 2011). Other studies also showed similar gender differences in body satisfaction (El Ansari et al., 2014; Griffiths et al., 2017; Inchley et al., 2020).

The strengths of this study are its relatively large sample size and its acceptable response rate. However, data were only collected within a single municipality in southern Sweden, which may limit the generalizability of the results. The desire was to capture a good representation. However, a university is placed in the included municipality and the education level in the municipality is higher than average. Also, the four schools which participated had more university preparatory courses, compared with the school who declined participating, which had more vocational study programs. These factors lead to a sample with a higher socio-economic status than average in Sweden. Furthermore, the study's cross-sectional design is unable to establish causality. It may be that adolescents who report a positive wellbeing tend to be more physically active and to have a positive body image. However, the converse may also be true: adolescents who are physically active and have a positive body image tend to have a better wellbeing. PA was self-reported and not objectively measured, which is another limitation. A high proportion of the adolescents in our study rated their wellbeing as very good or quite good and perceived their family finances to be good. A possible explanation for these results is that the sample may contain a disproportionate number of affluent individuals. However, similar results can be seen in the Swedish contribution to the HBSC study, which found that most 15-year-olds in Sweden rated their wellbeing as high or very high (Public Health Agency of Sweden, 2019).

## Conclusion

A positive body image, which include both body appearance and body function, and high PA levels were significantly associated with wellbeing in adolescents, which imply the importance of promotion of PA among young people. From a public health perspective, a better understanding of how PA can influence positive wellbeing may help inform politicians and school administrators and health care providers to incentivize children and adolescents to engage in PA both within and beyond the school's boundaries. Since physical self-concepts have been shown to be an even better predictor of behavior than characteristics such as height and weight, school PE together with school health care providers can promote positive body image and PA habits. What distinguishes our results from those of previous studies is that adolescent females in our sample reported positive wellbeing, positive body image, and PA levels equivalent and at times slightly higher than adolescent males, which can be demonstrated as a good example of non-specific gender behavior that can serve as a model for improved wellbeing among females. Approximately one-third of the participating adolescents did not rate their wellbeing as good. Further research is needed to determine the factors that can affect the wellbeing of adolescents to further improve health.

## Data Availability Statement

The raw data supporting the conclusions of this article will be made available by the authors, without undue reservation.

## Ethics Statement

The studies involving human participants were reviewed and approved by the Regional Ethical Review Board (EPN 2015/113). Written informed consent to participate in this study was provided by the participants' legal guardian/next of kin.

## Author Contributions

PG and A-CS contributed to the design of the study. PG contributed to data collection. A-CS, IS, and JF drafted the manuscript. All authors contributed to the interpretation of the data, revised the manuscript, provided final approval, and agreed to be accountable for all aspects of work to ensure integrity and accuracy.

## Funding

This work was sponsored by the Gyllenstiernska Krapperup Foundation and the Research Platform for Collaboration for Health, Faculty of Health Sciences at Kristianstad University, and Lund University.

## Conflict of Interest

The authors declare that the research was conducted in the absence of any commercial or financial relationships that could be construed as a potential conflict of interest.

## Publisher's Note

All claims expressed in this article are solely those of the authors and do not necessarily represent those of their affiliated organizations, or those of the publisher, the editors and the reviewers. Any product that may be evaluated in this article, or claim that may be made by its manufacturer, is not guaranteed or endorsed by the publisher.
